# The Composition of Circulating Leukocytes Varies With Age and Melanoma Onset in the MeLiM Pig Biomedical Model

**DOI:** 10.3389/fimmu.2020.00291

**Published:** 2020-02-28

**Authors:** Fany Blanc, Armelle Prévost-Blondel, Guillaume Piton, Edwige Bouguyon, Jean-Jacques Leplat, Fabrice Andréoletti, Giorgia Egidy, Emmanuelle Bourneuf, Nicolas Bertho, Silvia Vincent-Naulleau

**Affiliations:** ^1^INSERM, U1016, Institut Cochin, Paris, France; ^2^Université Paris-Saclay, INRAE, AgroParisTech, GABI, Jouy-en-Josas, France; ^3^CEA, DSV/iRCM/SREIT/LREG, Jouy-en-Josas, France; ^4^CNRS, UMR8104, Paris, France; ^5^Université de Paris, Paris, France; ^6^Université Paris-Saclay, INRAE, VIM, Jouy-en-Josas, France; ^7^CEA, DSV/iRCM/SREIT/LCE, Fontenay-aux-Roses, France; ^8^BIOEPAR, INRAE, ONIRIS, Nantes, France

**Keywords:** swine blood leucocytes, lymphoid cells, myeloid cells, longitudinal analysis, age, melanoma, biomedical model

## Abstract

Immunological research in pigs benefits from many improvements with a direct impact on the veterinary control of pig husbandry and on biomedical models. We compiled the available knowledge to develop gating strategies to monitor simultaneously all blood immune cell types by multicolor flow cytometry in Melanoblastoma-bearing Libechov Minipigs (MeLiM). The MeLiM pig spontaneously develops cutaneous melanomas that regress few months later. We monitored lymphoid and myeloid cell subsets in 3 to 21 weeks old pigs. Interestingly, neutrophils, type III monocytes (CD163^+^ CD14^+^ MHC II^−^) and CD4^−^ CD8α^−^ T cells are less abundant in oldest animals in contrast to eosinophils, type II monocytes (CD163^−^ CD14^low^ MHC II^+^), B cells, γδ T cells, CD4^+^ CD8α^+^ and CD4^−^ CD8α^+^ T cells. Melanoma occurrence led to changes in the blood cell composition. Higher proportions of NK cells, CD4^+^ and CD4^+^ CD8α^+^ T cells, and CD21^−^ B cells among B cells are found in young melanoma-bearing piglets, consistent with the immune-mediated spontaneous regression in the MeLiM model.

## Introduction

Immunological research in the porcine species is of great importance for its direct impact on the veterinary control of pig husbandry as well as for the development of biomedical models (1, 2). Melanoblastoma-bearing Libechov Minipigs (MeLiM) spontaneously develop cutaneous melanomas around birth, with clinical and histopathological features comparable to human counterparts (3, 4). Animals can carry multiple lesions from benign to highly invasive, eventually leading to lymph nodes and visceral metastasis. A spontaneous regression occurs between 2 and 4 months after birth, corresponding to a complete disappearance of tumors and metastasis, without any treatment. This regression process, often accompanied by a local or systemic depigmentation of hair, skin and eyes has been described in details, both clinically and histologically (4, 5). It is at least partially controlled by an efficient immune response toward melanoma cells (6).

Studies of porcine immune cells have been initially enabled by the development and characterization of monoclonal antibodies against porcine cluster of differentiation (CD) molecules as well as the identification of cross-reactive antibodies provided by three International Swine CD Workshops (7–9). Since then, efforts have been made to provide new tools to study immune cells (10–13). In addition, the use of omics analyses can help identifying molecular patterns shared between cell types across tissues and species, thus refining surface phenotyping as it was published for phagocyte mononuclear cells (14–16).

Most publications regarding tools specific to immunomonitoring in pigs focus on certain cell subtypes and do not consider studying all blood cell subsets simultaneously. Lymphoid cells are the most studied and current gating strategies allow proper identification of B cells, NK, NKT, γδ T cells, CD4, and CD8 T cells (11, 17, 18). Concerning the myeloid lineage, characterization of circulating conventional dendritic cells (DCs) by flow cytometry allowed a proper identification of swine cDC1 and cDC2 subsets (15, 19, 20). Blood monocytes identified in peripheral blood mononuclear cells (PBMCs) are heterogeneous. Most research focuses on the CD163 marker as it was found to be the entry point of the porcine reproductive and respiratory syndrome virus into macrophages. CD163 distinguishes two major populations of monocytes (21) that could be further divided into four subsets according to CD14 and MHC II expressions (22–24). Finally, granulocytes have not been much studied in swine blood. Indeed, the use of PBMCs in many studies may bias results due to the loss of particular populations (i.e., granulocytes) during cell preparation. The antibody clone PG68A has been described as a pan granulocyte marker, but its target is still unknown (9).

The aim of this study was to monitor all cell types simultaneously in swine peripheral blood leukocytes (PBLs). We thus compiled the available knowledge to develop gating strategies for multicolor flow cytometry. Four panels with lineage, exclusion and activation markers were used to identify lymphoid (αβ and γδ T cells, B cells, NK, and NKT cells) and myeloid cells (DCs, monocytes, granulocytes). The subsets of T lymphocytes were also distinguished according to their helper, cytotoxic or regulatory functions. We monitored MeLiM pigs from 3 to 21 weeks of age to gain knowledge on the maturation of pig's immune system before weaning until puberty and to evaluate the impact of melanoma occurrence in this biomedical model.

## Materials and Methods

### Experimental Pigs

Animal care and use was carried out under licenses issued by the Direction Départementale de la Protection des Populations des Yvelines (DDPPY, agreement number C78–719) and experiments were ethically approved by the Committee on the Ethics of Animal Experiments of AgroParisTech and INRA Jouy-en-Josas (COMETHEA, authorization number 12/091). Blood samples (5 mL) were collected on EDTA-containing Vacutainers from MeLiM pigs after puncture of the jugular vein. Fourteen males and 22 females were sampled 3–6 times (mean = 4.7) from 3 to 21 weeks of age. Samples were then allocated to eight groups of age (3–4, 5–6, 7–8, 9–10, 11–12, 13–15, 16–18, and 19–21 weeks). Pigs were individually examined for the presence of cutaneous melanoma every 1–2 weeks until 3 months of age and then every 2–3 weeks until 5 months. Among these 36 pigs, 29 animals bore a melanoma. The tumor burden was considered as low or high depending on the number of cutaneous melanoma that developed on each animal (low: 1–4; high: 5–14). The presence of palpable lymphadenopathies (lymph node adenomegalies) was also examined. Seven animals, melanoma free at the sampling time, were classified as healthy. The number of pigs sampled for each group of age with their sex, melanoma occurrence, tumor burden, and presence of palpable lymphadenopathies is reported in Table 1. Studied animals have never been vaccinated. The herd was serologically tested free of brucellosis, classical swine fever, porcine reproductive and respiratory syndrome virus and Aujeszky's disease. In addition, no diarrhea nor pneumonia were observed during the study. Weaning is progressive in the herd as piglets can start eating dry food before being separated from the sow (separation occurring between 5 and 8 weeks). Some blood samples were also collected on dry-Vacutainers and after centrifugation (350 *g*, 15 min, 20°C), sera were stored at −80°C.

**Table 1:** Number of pigs sampled for each group of age, with their sex, melanoma occurrence, presence of palpable lymphadenopathies, and tumor burden.

	**Age in weeks**								**Total blood samples**
	**3–4**	**5–6**	**7–8**	**9–10**	**11–12**	**13–15**	**16–18**	**19–21**	
All animals (*n* = 36)	22	14	32	14	22	26	16	15	161
Males (*n* = 14)	10	4	13	6	10	8	5	6	62
Females (*n* = 22)	12	10	19	8	12	18	11	9	99
Healthy pigs (*n* = 7)	7	1	7	3	5	2	2	2	29
Melanoma-bearing pigs (*n* = 29)	15	13	25	11	17	24	14	13	132
With palpable lymphadenopathies	3	10	21	9	11	21	11	10	96
With high tumor burden	8	10	18	6	9	20	10	10	91

### Preparation of Peripheral Blood Leukocytes

Absolute number and viability of cells were determined with the ViaCount Assay performed on easyCyte 6HT-2L Guava flow cytometer (Millipore) following manufacturer's instructions. Red blood cells were lysed by transferring blood into a Tris-NH_4_Cl 140mM, pH 7.5 solution and incubated at room temperature for 20 min. Cells were then centrifuged (300 *g*, 5 min, 20°C) and a second red blood cell lysis was performed if necessary. Cells were thereafter washed twice in phosphate buffered saline (PBS) containing 5% fetal bovine serum (FBS) and cell number and viability were again determined in PBLs with the ViaCount Assay. One to five millions of PBLs were processed in each staining combination.

### Flow Cytometry

PBLs were first stained for viability with the aqua LIVE/DEAD® Fixable Dead Cell Stain Kit (Thermo Fisher Scientific) following manufacturer's instructions and further incubated for 20 min at 4°C in a blocking buffer (PBS containing 5% FBS, 5% horse serum, and 5% porcine serum). Cells were then centrifuged (300 *g*, 5 min, 4°C) and surface staining was performed in three steps. The four combinations of antibodies used are listed in Table 2 (combinations A-D). PBLs were first incubated for 20 min with a combination of primary antibodies of different mouse isotypes. After a wash in PBS containing 5% FBS, the appropriate combination of secondary antibodies specific for mouse immunoglobulin (Ig) subclasses labeled with fluorochromes was incubated for 20 min to the cell pellets. Cells were washed in PBS containing 5% FBS and finally incubated for 20 min with directly labeled primary antibodies. Detection of CD79a or Foxp3 was done by intracellular staining using Foxp3/Transcription Factor Staining Buffer Set (eBioscience) with a slight modification of the protocol recommended by the manufacturer. Briefly, samples were incubated for 2 h in fixation/permeabilization buffer, washed and incubated for 2 h with labeled antibodies diluted in permeabilization buffer. Foxp3 and CD79a intracellular staining specificity was determined by incubating cells with PE-labeled isotype controls (rat IgG2a or mouse IgG1, respectively). All antibodies used in this study were titrated for optimal signal/noise ratios as indicated in Table 2. Cells were then fixed in BD CellFIX solution before analysis on a BD LSR Fortessa cytometer (BD Biosciences). The data analysis was performed using FlowJo V10 software. Electronic compensation was used to eliminate residual spectral overlaps between individual fluorochromes.

**Table 2:** Antibodies used in this study.

**Specificities**	**Clones/references**	**Isotypes**	**Target species**	**Fluorochrome**	**Labeling strategy**	**Providers**	**Working dilutions or concentrations**	**Used in combinations**
CD45	K252- 1E4	m IgG1	p	AF647	Directly conjugated	1	1/20	A, B, C, D, E
CD3	PPT3	m IgG1	p	FITC	Directly conjugated	2	2.5 μg/mL	A, B
CD8alpha	76-2-11	m IgG2a	p	PE-Cy5	Directly conjugated	3	1 μg/mL	A, B
CD4	74-12-4	m IgG2b	p	APC-Cy7	Secondary antibody^a^	3	2.5 μg/mL	A, B
γδTCR	PGBL22A	m IgG1	p	PE-Cy7	Secondary antibody^b^	4	2 μg/mL	A
CD16	G7	m IgG1	p	PE	Directly conjugated	1	1/20	A
CD25	K231.3B2	m IgG1	p	PE-Cy7	Secondary antibody^b^	1	1/10	B
Foxp3*	FJK-16s	r IgG2a	m/r	PE	Directly conjugated	5	1/50	B
control	eBR2a	r IgG2a		PE	Directly conjugated	5	1/50	B
MHC II	MSA3	m IgG2a	p	AF488	Secondary antibody^c^	4	2 μg/mL	C, D
CD21	B-Ly4	m IgG1	h	PE-Cy7	Secondary antibody^b^	6	4 μg/mL	C
CD79a*	HM57	m IgG1	h	PE	Directly conjugated	1	50 μg/mL	C
control	MOPC-21	m IgG1		PE	Directly conjugated	7	1/20	C
PG68A	PG68A	m IgG1	p	PE-Cy7	Secondary antibody^b^	4	5 μg/mL	D
CD163	HM57	m IgG1	p	PE	Directly conjugated	1	1/10	D, E
CD172a	74-22-15A	m IgG2b	p	APC-Cy7	Secondary antibody^a^	4	1 μg/mL	D, E
CD14	TUK4	m IgG2a	h	Pacific Blue	Directly conjugated	1	1/10	D
CADM1	3E1	c IgY	h/m	Qdot655	Secondary antibody^f^	8	4 μg/mL	D
CD14	TUK4	m IgG2a	h	AF488	Biotin-streptavidin^e^	1	1/20	E
MHC II	MSA3	m IgG2a	p	PerCp-eFluor710	Secondary antibody^d^	4	2 μg/mL	E

A, B, C, and D correspond to the four antibodies combinations to identify cell subsets: A for lymphocyte subsets, B for Tregs, C for B lymphocytes, and D for myeloid cells (*intracellular stainings). Combination E was used to analyze monocytes by imaging flow cytometry. (1: AbD Serotec, 2: SouthernBiotech, 3: Abcam, 4: WSU, 5: eBioscience, 6: BD Biosciences, 7: Exbio, 8:MBL, c: chicken, h: human, m: mouse, p: pig, r: rat).

a *Goat anti-mouse IgG2b-APC-Cy7, 1.25 μg/mL, Abcam*.

b *Goat anti-mouse IgG1-PE-Cy7, 0.5 μg/mL, eBioscience*.

c *Goat anti-mouse IgG2a-AF488, 5 μg/mL, Invitrogen*.

d *Goat anti-mouse IgG2a-PerCp-eFluor710, 0.5 μg/mL, eBioscience*.

e *Streptavidin-AF488, 5 μg/mL, Invitrogen*.

f *Goat anti-chicken IgY-Qdot655, 2.5nM, Exbio*.

### Cell Sorting and May–Grünwald–Giemsa Staining

Myeloid cells were stained as described for flow cytometry analysis (Table 2, combination D), but were not fixed. They were sorted using a FACS Aria III cell sorter (BD Biosciences; 100 μm nozzle). Sorted cells were dropped off on microscope slides (Superfrost, Thermo) by centrifugation and incubated with May–Grünwald–Giemsa (MGG) stains. Images were acquired with a digital slide scanner (Pannoramic SCAN, 3DHISTECH), objective magnification X40 and visualized with the Pannoramic Viewer software.

### Imaging Flow Cytometry

Cells were stained as described for flow cytometry analysis (Table 2, combination E) without viability staining. Cells were finally fixed in 50 μL of BD CellFIX solution (BD Biosciences) and nuclei were stained by adding DAPI (1 μg/mL) and Triton X-100 (0.1%). Images were acquired on an Amnis ImageStreamX imaging flow cytometer (Merck Millipore) with the 60X objective. Data were analyzed using IDEAS 6.2 software following a method adapted from Pelletier et al. (25). Monocyte subpopulations were gated among in-focus single cell events, using the same gating strategy as for flow cytometry analysis. Average measurements from multiple masks selected after visual inspection were used to accurately represent the cell surface (bright field area in Ch01, 3 masks) and nuclei (DNA dye intensity in Ch07, 4 masks). These average cellular and nuclear areas were used to create a combined feature to calculate the “per cell” average cytoplasmic area (area of the cell reduced by the area of the nucleus) and N/C ratio (average nuclear area divided by the average cytoplasmic area).

### IgG ELISA

Total IgGs sera concentrations were measured with the Pig IgG ELISA Quantification Kit (Bethyl Laboratories). Samples were tested at 1–100,000 or 250,000 dilutions. Assays were performed in duplicate and according to manufacturer's instructions.

### Statistical Analysis

Absolute cell counts were used to calculate absolute numbers of various subpopulations. Statistical analyses were performed taking into account the age group, sex and melanoma occurrence of pigs. For statistical inference, logarithm or square root transformations were applied to the data expressed in absolute count or percentages, respectively. A linear regression model was then applied using the lm() function of R (version 3.6.0) (26) on transformed data with sex, age group and melanoma occurrence as fixed effects. Likelihood ratio tests were performed to evaluate the effects of sex, age group or melanoma occurrence for each cell subset and data are reported in Table 3 and Supplementary Tables 1–3. When an effect was significant, analysis of variance followed by Tukey's *post-test* was performed to compare the groups. Results in the text are expressed as mean +/– SD of the raw data and figures represent the plot of the raw data. Other statistical tests used are mentioned in the appropriate figure legend.

**Table 3:** Absolute numbers and proportions of lymphocytes, monocytes and granulocytes in pig blood, and effect of age, sex, and melanoma occurrence on those phenotypes.

**Cells (phenotype)**	**Mean**	**SD**	**95% CI of mean**		**Unit**	**(*****p*****) effects of**		
			**Lower**	**Upper**		**Age**	**Sex**	**Melanoma**
**Leukocytes**	13.8	6.2	12.8	14.8	10^6^ cells / mm^3^	**<0.001**	0.824	0.470
**Lymphocytes**	4.8	2.1	4.5	5.1	10^6^ cells / mm^3^	**0.012**	0.999	**0.022**
(FSC^low^ SSC^low^)	36.9	11	35.2	38.6	% of PBLs	**<0.001**	0.614	**0.020**
**Monocytes**	1.7	1	1.6	1.9	10^6^ cells / mm^3^	**<0.001**	0.406	0.528
(FSC^med^ SSC^med^)	12.1	2.8	11.7	12.6	% of PBLs	0.208	**0.015**	0.998
**Granulocytes**	7.3	4.5	6.6	8	10^6^ cells / mm^3^	**<0.001**	0.444	0.853
(FSC^med^ SSC^high^)	51.0	10.9	49.3	52.7	% of PBLs	**<0.001**	0.160	**0.031**

*Means, SD and 95% CI of mean obtained for the 36 pigs sampled from 3 to 21 weeks (n = 161) of lymphocytes, monocytes and granulocytes subpopulations gated basically by their size and morphology properties (being FSC^low^ SSC^low^, FSC^med^ SSC^med^, and FSC^med^ SSC^high^, respectively). Effects of age, sex and melanoma occurrence were calculated by likelihood ratio tests. All significant results are in bold*.

## Results

### Longitudinal Analysis of Global Immune Cells in Swine Blood: Decrease in Granulocyte and Increase in Lymphocyte Proportions With Age

**Figure 1:**
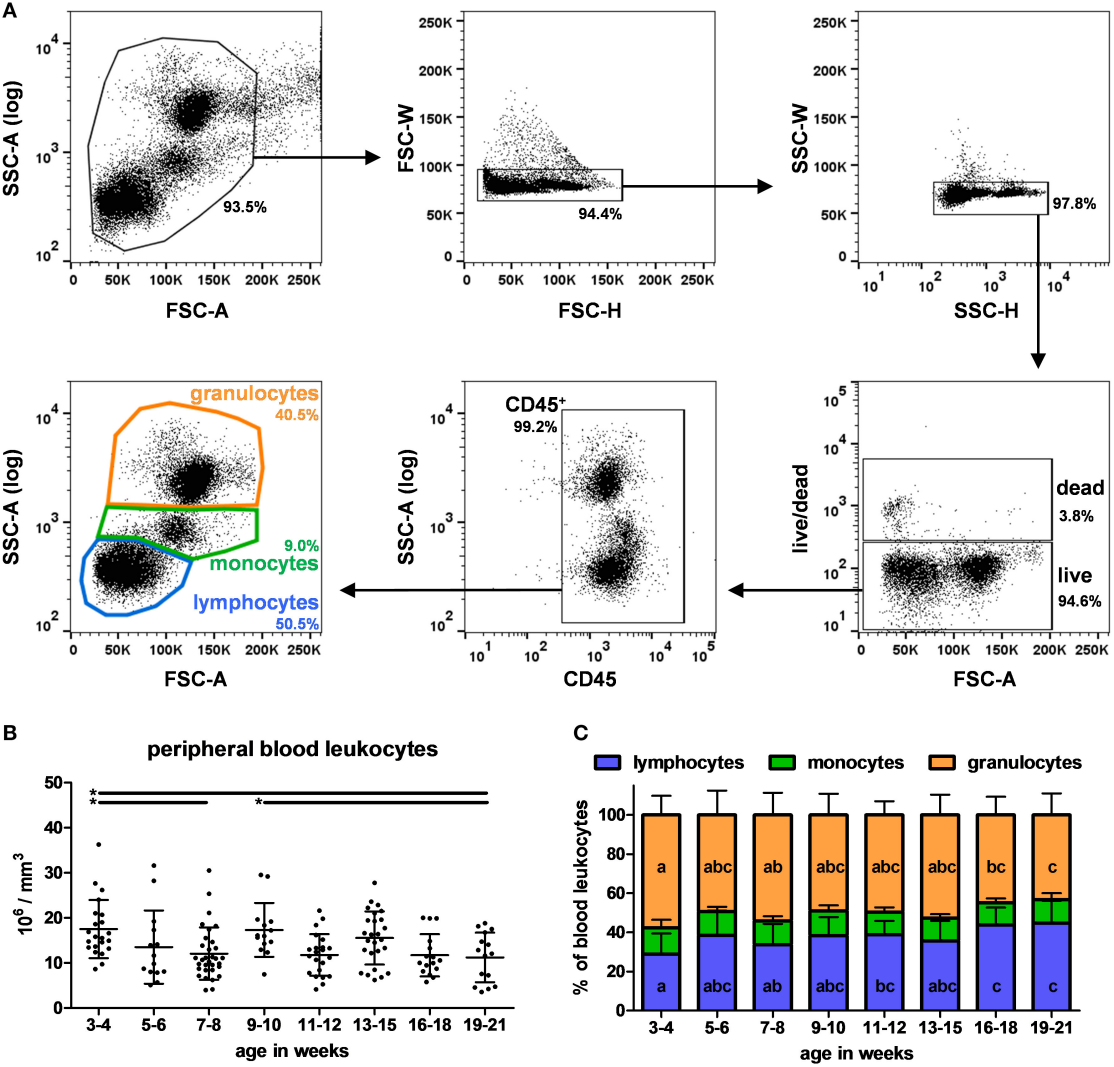
Identification and quantification of lymphocytes, monocytes and granulocytes in swine peripheral blood. **(A)** Illustrative dot plots showing the gating strategy used to identify PBLs subsets. Cells were first gated on FSC-A vs. SSC-A dot plot (upper left panel) and doublets were excluded on FSC-H vs. FSC-W dot plot (“singlets-1,” upper middle panel) and SSC-H vs. SSC-W dot plot (“singlets-2,” upper right panel). Dead cells were then excluded using live/dead staining (lower right panel). Immune cells were stained for CD45 (lower middle panel). Lymphocytes, monocytes, and granulocytes subpopulations from CD45^+^ cells were finally gated using size and morphology properties (lower left panel). Percentages of the parent populations are shown on each dot plot representing 10,000 events. **(B)** Absolute leukocyte count in blood according to pigs' age. Lines represent the means with SEM. Significant differences within groups of age are represented with bars (*p < 0.05). **(C)** Lymphocytes, monocytes and granulocytes distribution according to pigs' age. Different letters inside the bar plots indicate significant differences within groups of age for the corresponding cell population (*p* < 0.05).

The different cell subpopulations in peripheral blood from 3 to 21 week-old pigs were analyzed by flow cytometry using four combinations of antibodies applied to each sample (A-D listed in Table 2) and a gating strategy illustrated in Figure 1A. All proportions and absolute numbers of cell subsets are reported in Table 3. Total leukocyte count was 13.8 +/– 6.2 millions per mm^3^ with a high dispersion at each time point between animals (Figure 1B). On average, lymphocytes, monocytes and granulocytes represented 36.9% +/– 11.0, 12.1% +/– 2.8 and 51.0% +/– 10.9 of PBLs, respectively (Figure 1C). Of note, the proportion of granulocytes decreased in oldest pigs (effect of age *p* < 0.001 and *p* < 0.01 comparing 3–4 weeks to 16–18 and 19–21 week-old groups, respectively). Differences were also observed in absolute count. On the other hand, the proportion of lymphocytes increased at the same time points (effect of age *p* < 0.001). The proportion of monocytes varied among pigs (with 3 to 4-fold differences between minimum and maximum), but no effect of age was observed over the 16 weeks period.

### Longitudinal Analysis of Swine Blood NK, NKT, γδ T Cells, CD4 and CD8 T Cells, and Tregs: High Proportion of γδ T Cells and Increase in CD4^+^ CD8α^+^ T Cells With Age

**Figure 2:**
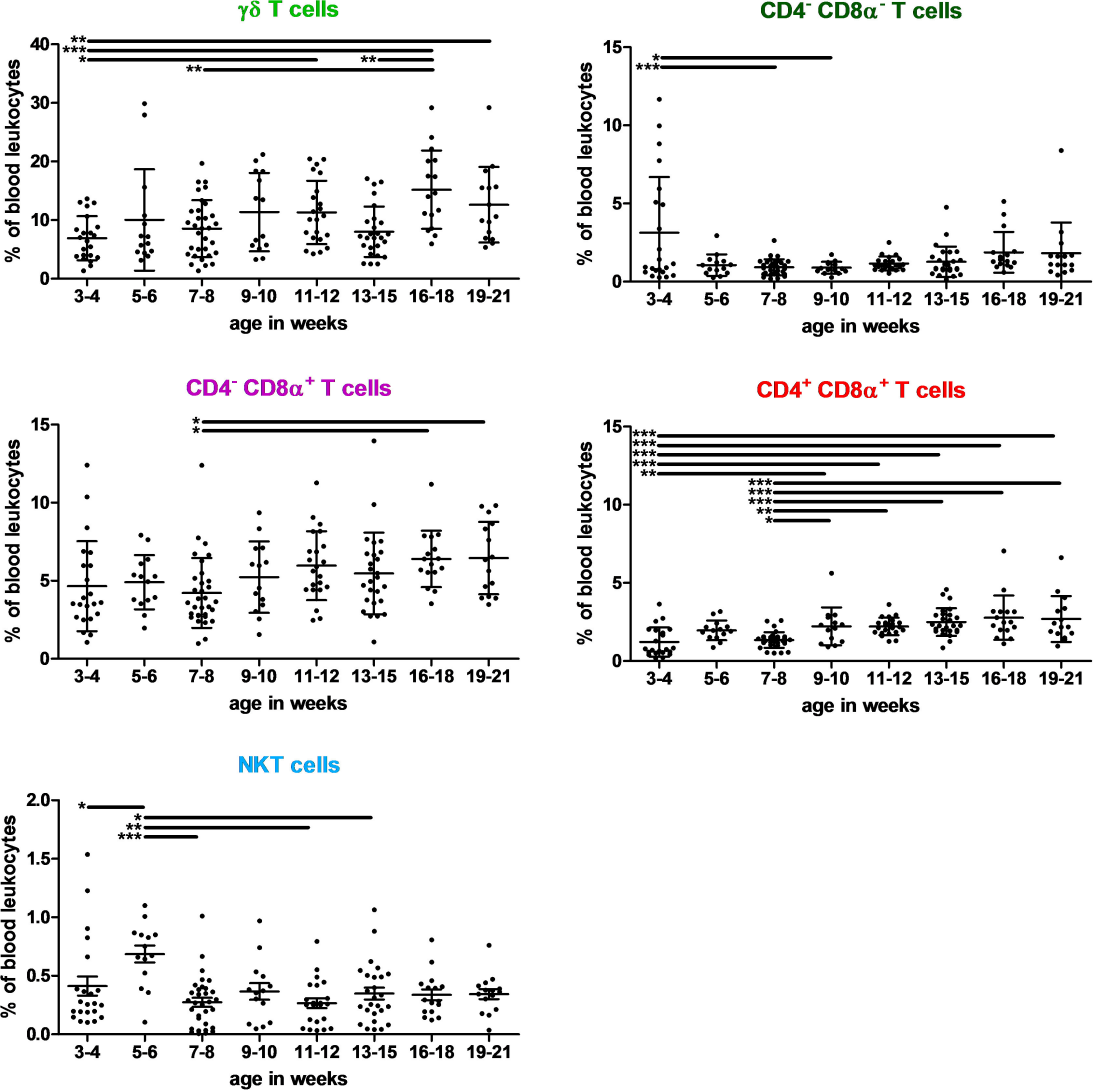
Kinetics of percentages of γδ T, CD4^−^ CD8α^−^, CD4^−^ CD8α^+^ T, CD4^+^ CD8α^+^ T, and NKT lymphocytes in swine peripheral blood. Lines represent the means with SEM. Significant differences are represented with bars (*p < 0.05, **p < 0.01, and ***p < 0.001).

The phenotypes and absolute numbers of NK, NKT, γδ T cells, CD4, and CD8 T cells, and Tregs were investigated in the blood of pigs using the antibody panels A and B, listed in Table 2. The gating strategy is presented in Supplementary Figure 1A. Supplementary Table 1 reports the different proportions and numbers found for those subsets and their evolution between 3–4 and 19–21 weeks of age are presented in Figure 2 (subsets varying over time) and Supplementary Figure 1B (stable subsets over time).

No significant differences among PBLs were observed with age for NK (3.2% +/– 2.3), CD4^+^ T helper cells (6.6% +/– 3.3) and Tregs (1.0% +/– 0.3). Most of these latter were CD4^+^ (63.7%), whereas CD4^+^ CD8α^+^ (double positive cells, or DP) and CD8α^+^ represented 16.8 and 12.5% of Tregs, respectively. Only DP Tregs slightly increased in oldest pigs (data not shown).

Conversely, differences with age were observed for the following subsets: NKT cells represented only 0.4% of PBLs, whereas γδ T cells represented 9.9% of them and were the most abundant T cells. DP T cells (*p* < 0.001) represented only 1.2% of PBLs at 3–4 weeks of age, remained at that level until 7–8 weeks and then reached 2.7% of PBLs in 16 to 21 week-old pigs. Cytotoxic T cells represented 5.2% +/– 2.4 of PBLs. A slight increase in cytotoxic T cell levels was observed with age (*p* = 0.002) with lower levels for 7–8 week-old pigs. Finally, CD4^−^ CD8α^−^ (double negative cells, or DN) T cells represented <2% of PBLs in most cases. Nevertheless, they could represent 11.7% of PBLs and 38.7% of T cells in 3–4 weeks old piglets, with a very high heterogeneity.

### Progressive Increase of B Cells and Serum IgG Levels With Age While CD21 Decreased

**Figure 3:**
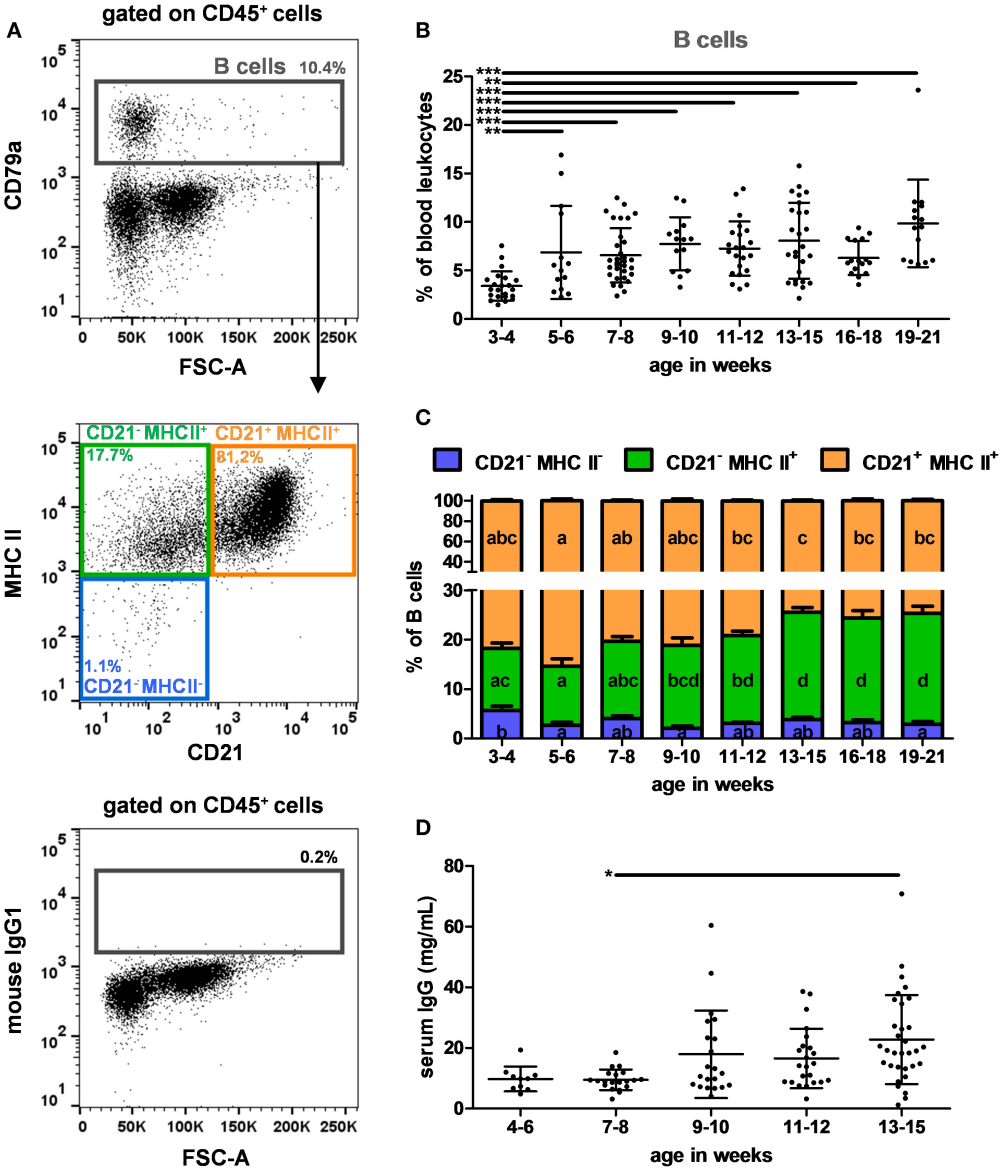
B cells in swine peripheral blood. **(A)** Gating strategy to identify B cells in PBLs. B cells were identified as CD79a^+^ among the CD45^+^ population described in Figure 1A (upper panel). A representative dot plot with CD79a isotype control (mouse IgG1) is shown (lower panel). CD21 and MHC II expression of B cells were studied (middle panel). Percentages of the parent populations are shown on each dot plot representing 10,000 events. Kinetics of the percentages of **(B)** B cells in PBLs and **(C)** CD21 and MHC II expressions among B cells. **(D)** Kinetics of total IgGs in sera assessed by ELISA. Lines represent the means with SEM. Significant differences within groups of age are represented with bars (*p < 0.05, **p < 0.01, and ***p < 0.001) or with different letters inside the bar plots of the corresponding population (*p* < 0.05).

To identify B cells, CD79a remained the best marker (27), as the anti-human CD19 antibody (clone B-D3) reported to cross-react with porcine B cells (28) did not label any cells in our experiments (data not shown). Therefore, B cells were identified using the antibody panel C (listed in Table 2) and the gating strategy illustrated in Figure 3A. To illustrate the specificity of the intracellular CD79a staining, the FMO control is depicted. Different B cell subsets were also analyzed according to their CD21 and MHC II expressions. On average, CD79a^+^ B cells represented 6.9% +/– 3.6 of PBLs (Supplementary Table 2). Interestingly, they were significantly less abundant in 3–4 week-old piglets (3.4% of PBLs) than in oldest ones (6.3 and 9.9% of PBLs, *p* < 0.001) (Figure 3B). We observed that the CD21 expression on B cells decreased with age (*p* < 0.001) (Figure 3C). The serum IgG levels were also analyzed to evaluate the humoral response (Figure 3D). They remained low until 7–8 weeks and increased thereafter (effect of age, *p* = 0.012) with a significant difference between 7–8 and 13–15 week-old pigs (*p* = 0.01). Serum IgG levels significantly correlated with B cells (expressed in absolute number and in percentage of PBLs, *n* = 78, Pearson's correlations: *p* = 0.016 and 0.019, with *r* = 0.272 and 0.265, respectively), and with the CD21^−^ MHC II^+^ B cell subset (expressed in absolute number and in percentages of PBLs or B cells, *n* = 78, Pearson's correlations: *p* < 0.001, *p* = 0.002 and *p* = 0.018 with *r* = 0.371, 0.350, and 0.268, respectively).

### Longitudinal Analysis of Swine Blood DCs and Monocytes: Rapid Decrease of CD163^+^ CD14^+^ MHC II^−^ Monocytes and Increase of CD163^−^ Monocytes With Age

**Figure 4:**
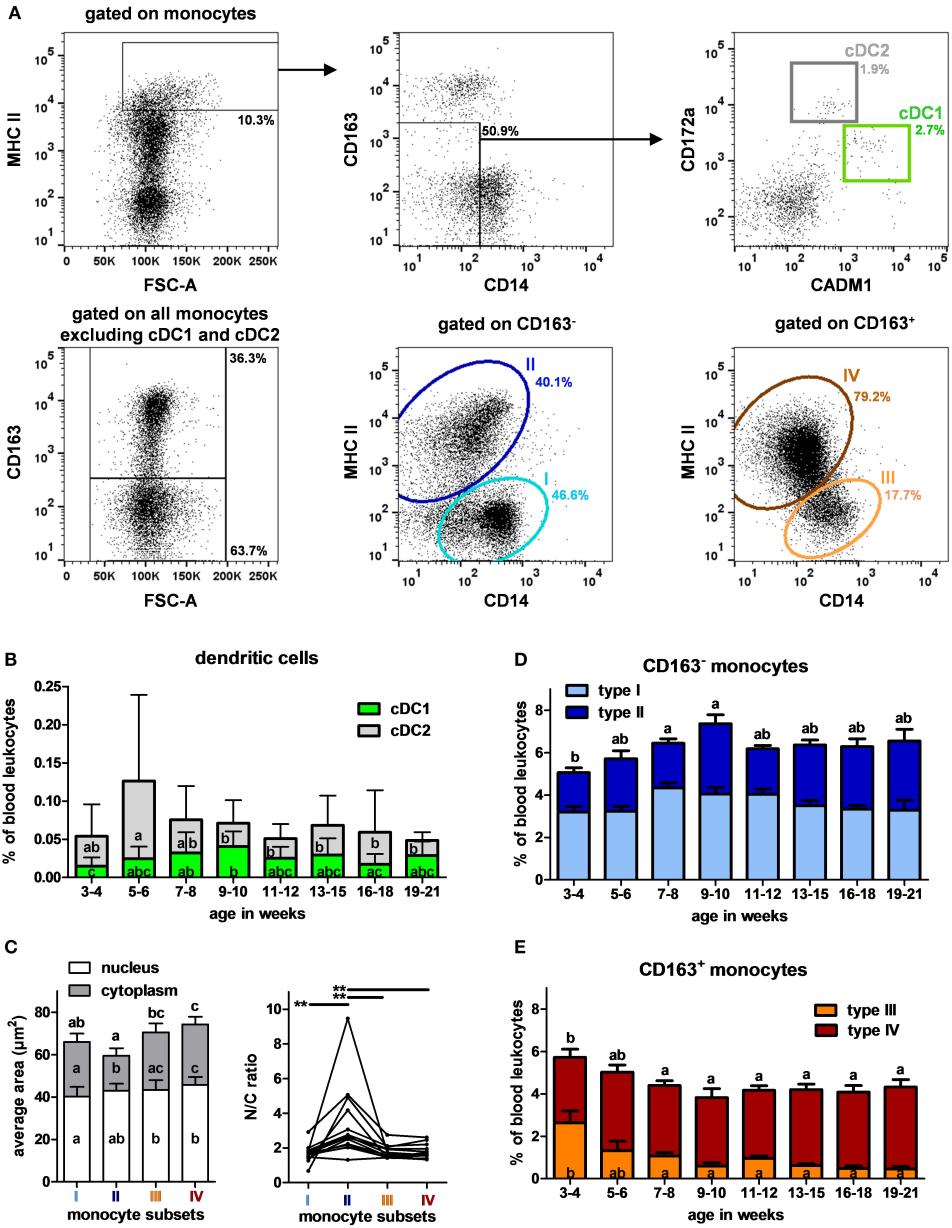
DCs and monocytes in swine peripheral blood. **(A)** Gating strategy to identify cDC1, cDC2 and monocytes in PBLs. Cells were first gated on the monocyte cell population described in Figure 1A. Within monocytes, MHC II^high^ were selected (upper left panel) and CD14^−/low^ CD163^−/low^ were gated (upper middle panel) and plotted on CADM1 vs. CD172a (upper right panel). cDC1 and cDC2 were gated as CADM1^high^ CD172a^low^ and CADM1^low^ CD172a^high^, respectively. A Boolean gate was then created to get all the monocytes excluding only cDC1 and cDC2. Cells were then separated in CD163^+^ and CD163^−^ (lower left panel) and MHC II and CD14 expressions were evaluated within those cell subsets. Within CD163^−^ monocytes (lower middle panel), type I (CD163^−^ CD14^+^ MHC II^−^) and type II (CD163^−^ CD14^low^ MHC II^+^) were identified and within CD163^+^ monocytes (lower right panel), type III (CD163^+^ CD14^+^ MHC II^−^) and type IV (CD163^+^ CD14^low^ MHC II^+^). Percentages of the parent populations are shown on each dot plot representing 10,000 events. **(B)** Kinetics of percentages of cDC1 and cDC2 in peripheral blood. Lines represent the means with SEM. Different letters inside the bar plots indicate significant differences within groups of age for the corresponding cell population (*p* < 0.05). **(C)** Per cell nuclear and cytoplasmic areas and N/C ratios (nuclear divided by cytoplasmic area) of the monocyte subsets assessed by imaging flow cytometry. Lines link data from the same animals. Statistical analysis was performed using a Friedman test for non-parametric repeated values followed by Dunn's post test to compare the different monocyte subsets. Significant differences within monocyte subsets are represented with bars (**p < 0.01) or with different letters inside the bar plots of the corresponding population (*p* < 0.05). Letters above the bar plots indicate the significant differences for the total cell area. Kinetics of percentages of **(D)** CD163^−^ and **(E)** CD163^+^ monocytes subsets in peripheral blood. Lines represent the means with SEM. Different letters inside the bar plots indicate significant differences within groups of age for the corresponding population (*p* < 0.05), letters above indicate the significant differences within groups of age for all the CD163^−^ or CD163^+^ monocytes (*p* < 0.05).

Recent studies have reported combinations of antibodies to identify circulating DCs in pig (15, 19, 20), cDC1 being CD14^−^ CD172a^low^ CADM1^+^ and cDC2 CD14^−/low^ CD172a^high^ CADM1^low^. Monocytes were described in pigs based on their expression of CD163, CD14, and MHC II (24). Accordingly, a specific combination (Table 2, panel D) was set up to analyze myeloid cells with the gating strategy illustrated in Figure 4A. cDC1 and cDC2 represented 0.03 and 0.04% of PBLs, respectively (Supplementary Table 3). Their proportions did not vary significantly with age except that cDC1 and cDC2 were more abundant at 9–10 and 5–6 weeks (*p* < 0.01), respectively (Figure 4B).

The characterization of monocytes was performed from the gate excluding cDC1 and cDC2 (Figure 4A). CD163 expression allowed to separate monocytes into two populations that could be further divided into 4 subsets according to their CD14 and MHC II expressions: CD163^−^ CD14^+^ MHC II^−^ (type I), CD163^−^ CD14^low^ MHC II^+^ (type II), CD163^+^ CD14^+^ MHC II^−^ (type III), and CD163^+^ CD14^low^ MHC II^+^ (type IV) (23, 24). Means of FSC were weaker for CD163^−^ monocytes than for CD163^+^ monocytes. To go further on the evaluation of their morphologic features, monocytes from pigs of 6–7 (*n* = 3), 11–12 (*n* = 9) and 16–18 (*n* = 2) weeks of age were studied by imaging flow cytometry. Areas of cells, nucleus and cytoplasm and the nucleus to cytoplasm ratios were evaluated “per cell” for the different subsets. Type II monocytes appeared morphologically different from others, harboring smaller cytoplasmic area and higher nucleus to cytoplasm ratios. Type III and IV (CD163^+^) were the biggest ones (Figure 4C).

The proportions of CD163^−^ monocytes increased until weeks 9–10 and then remained stable, representing around 6% of PBLs, type I and type II monocytes having the same evolution profile (Figure 4D). Type IV monocytes remained stable over time. In contrast, type III monocytes are more abundant in the youngest studied animals (effect of age *p* < 0.001) (Figure 4E). A high variability in the proportions of type III monocytes is observed within pigs between 3 and 6 weeks of age. After 13 weeks, type I, II, III and IV monocytes represented <5, 24–27, 26–30, and 30–32% of total monocytes, respectively.

### Longitudinal Analysis of Circulating Granulocytes in 3–21 Week-Old Pigs: Inverse Correlation Between the Proportion of Neutrophils and Eosinophils With Age

**Figure 5:**
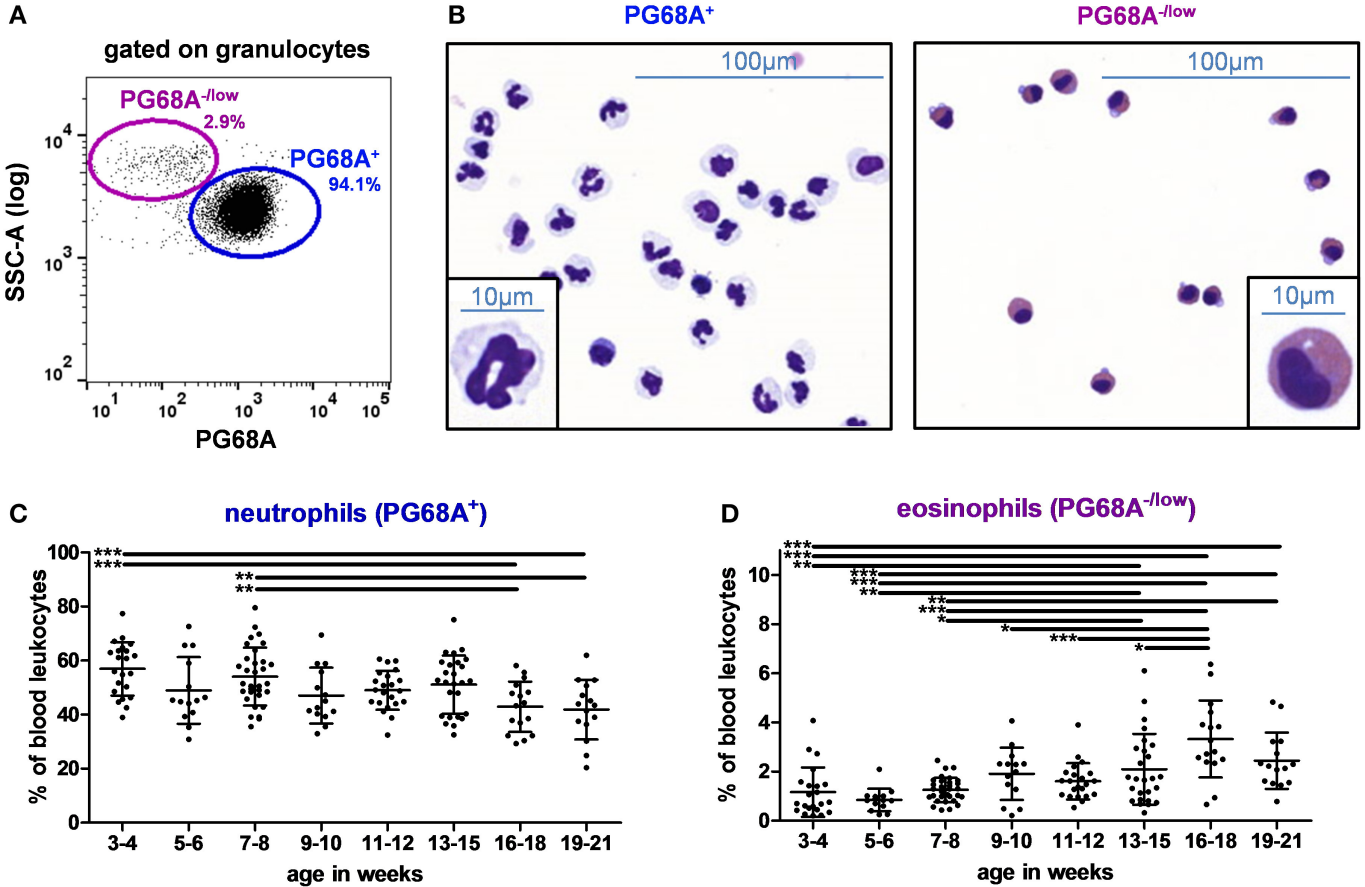
Granulocytes in swine peripheral blood. **(A)** Gating strategy to identify granulocytes in PBLs. Cells were first gated on the granulocyte population described in Figure 1A. PG68A^−/low^ and PG68A^+^ cells were identified. Percentages of the parent populations are shown on each dot plot representing 10,000 events. **(B)** Microscopic images (MGG staining, × 40 original magnification or × 100 in the insert) of sorted PG68A^+^ and PG68A^−/low^ cells. Images are representative of 3 independent sortings performed on 15 to 21 week-old pig granulocytes. Kinetics of percentages of **(C)** neutrophils and **(D)** eosinophils in peripheral blood. Lines represent the means with SEM. Significant differences are represented with bars (*p < 0.05, **p < 0.01, and ***p < 0.001).

To analyze granulocytes, the antibody clone PG68A was used (Table 2, panel D) and the gating strategy illustrated in Figure 5A was applied. Most of granulocytes expressed the PG68A marker, but some were PG68A^−/low^. These two subsets were then sorted and stained by MGG. As shown in Figure 5B, 94% of PG68A^+^ granulocytes had features corresponding to neutrophils with a multilobed nucleus and a cytoplasm containing few organelles and harboring a neutral pink stain. By contrast, 86% of PG68A^−/low^ granulocytes exhibited specific features of eosinophils with two lobes to their nucleus and acidophilic granules in their cytoplasm stained bright red, or reddish-purple. PG68A^+^ (mainly neutrophils) represented 56.9 and 54.1% of PBLs at 3–4 and 7–8 weeks, respectively; and decreased to 42.9 and 41.8% of them at 16–18 and 19–21 weeks, respectively (Supplementary Table 3 and Figure 5C, effect of age *p* < 0.001). PG68A^−/low^ (mainly eosinophils) were rare in young piglets and increased with age, representing 1.2, 0.9, and 1.3% of PBLs at 3–4, 5–6, and 7–8 weeks, respectively and 3.3 and 2.4% of PBLs at 16–18 and 19–21 weeks, respectively (Supplementary Table 3 and Figure 5D, effect of age *p* < 0.001).

### Influence of the Gender on Blood Cell Composition

Next, the effect of sex on the blood leukocyte composition was investigated from 62 samples derived from 14 males and 99 samples from 22 females. Globally, slight differences could be revealed: females harbored a higher proportion of type IV monocytes, CD4^+^ T helper cells and a lower proportion of CD8α^+^ cytotoxic T cells and cDC2 (see effect of sex in Table 3 and Supplementary Tables 1, 3).

### Effect of Melanoma Occurrence on the Blood Cell Composition in the MeLiM Model

**Figure 6:**
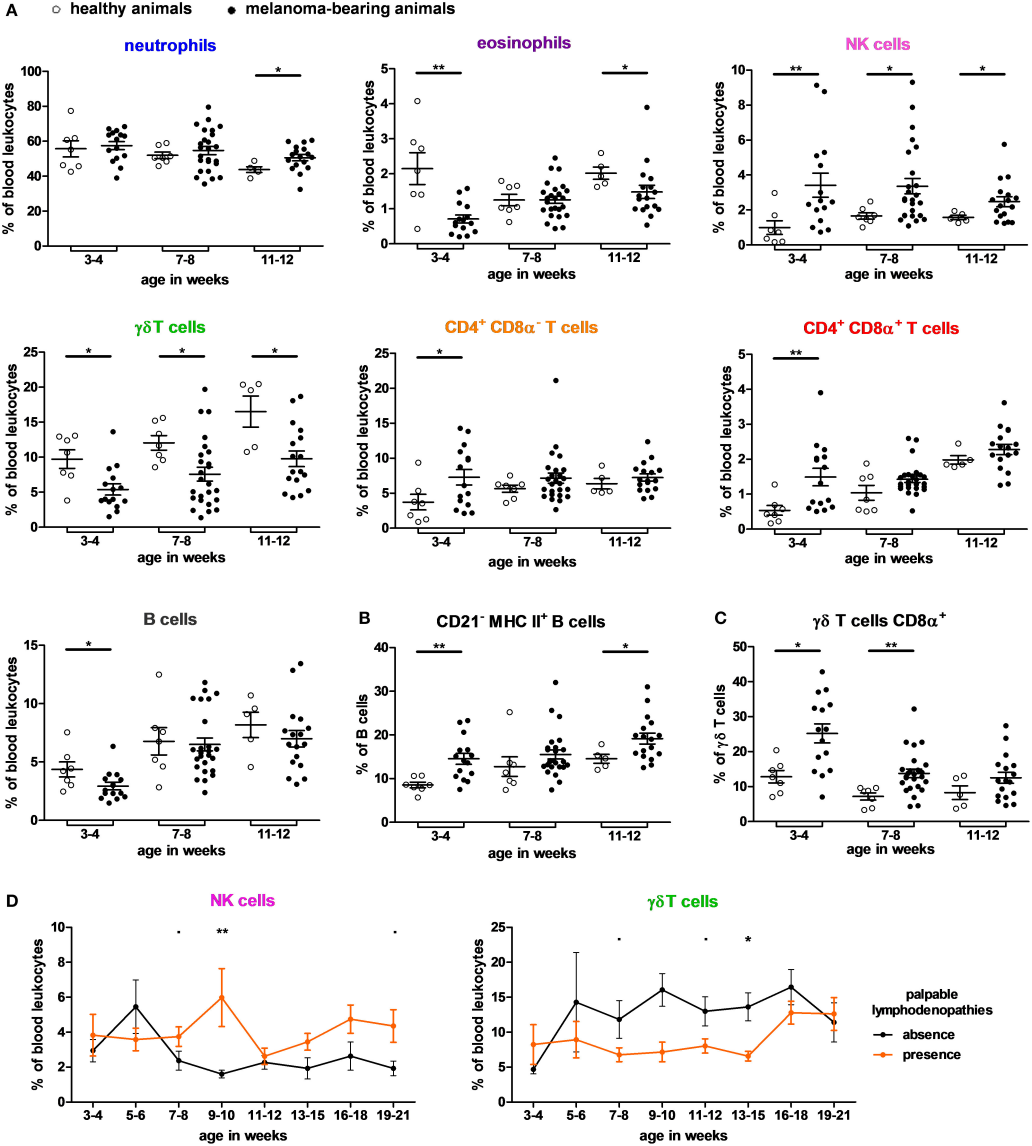
Effect of melanoma occurrence on immune blood cell composition of MeLiM pigs. **(A)** Blood cell proportions that differ between healthy and melanoma-bearing pigs aged of 3–4, 7–8, and 11–12 weeks. Proportions **(B)** of B cells CD21^−^ MHC II^+^ among B cells and **(C)** of γδ T cells expressing CD8α among γδ T cells from healthy and melanoma-bearing pigs aged of 3–4, 7–8, and 11–12 weeks. Healthy and melanoma-bearing pigs at each age were compared using a Mann-Whitney test and significant differences are represented with bars (*p < 0.05 and **p < 0.01). **(D)** Kinetics of proportions of NK cells and γδ T cells in blood leukocytes depending on the presence of palpable lymphadenopathies and tumor burden in melanoma-bearing pigs (means with SEM). Groups of pigs at each age were compared using a Mann-Whitney test and significant differences are represented above (*p* < 0.1 and *p < 0.05).

In this analysis, 29 samples came from 7 healthy pigs and 132 samples from 29 melanoma-bearing pigs. The effect of melanoma occurrence was then investigated on blood cell composition. Globally, there was no effect of melanoma on cDC1 and monocyte subsets. Melanoma-bearing animals harbored higher proportions of neutrophils, NK cells, cDC2, CD4^+^, and DP T cells; and lower proportions of eosinophils, γδ T cells and B cells (see effect of melanoma in Supplementary Tables 1–3). The differences were observed over three different periods related with important time points of melanoma development and then regression (at 3–4 weeks: tumor progression; at 7–8 weeks, first histological features of regression often observed; and at 11–12 weeks: more advanced features of regression) (4). Individual data for each cell subset in percentages of PBLs and in absolute counts are represented in Figure 6A and Supplementary Figure 2, respectively.

Neutrophils were more frequent in melanoma-bearing animals only at the latest time-point (11–12 weeks). Interestingly, 3–4 week-old melanoma-bearing pigs harbored higher proportions of NK cells and single positive CD4 or DP T cells and lower proportion of B cells, eosinophils and γδ T cells compared to age-matched healthy pigs. These differences between both groups were even observed all along the follow-up for NK cells and γδ T cells and were also consistent with absolute counts in most of cases (Supplementary Figure 2). Indeed, our data evidenced a significant accumulation of circulating NK cells and single positive CD4 or DP T cells in very young melanoma bearing pigs.

Notably, even though γδ T cells and B cells were less frequent in melanoma-bearing pigs, there were higher proportions of CD21^−^ MHC II^+^ B cells (Figure 6B) and CD8α^+^ γδ T in these animals (Figure 6C).

An effect of the presence of palpable lymphadenopathies and to a lesser extent of tumor burden can be found on the proportions of γδ T cells and NK cells in blood; the presence of palpable lymphadenopathies leading to higher proportions of NK cells and lower of γδ T cells (Figure 6D).

## Discussion

In this study, we analyzed phenotypic and quantitative changes in blood immune cell subpopulations before weaning until puberty to provide an overview of the maturation of the pig immune system in the biomedical MeLiM model. We determined relevant strategies to monitor all circulating subsets simultaneously. Lymphoid and myeloid cells were phenotyped and functional subsets for T lymphocytes (helper, cytotoxic or regulatory functions) and B lymphocytes were characterized.

**Figure 7:**
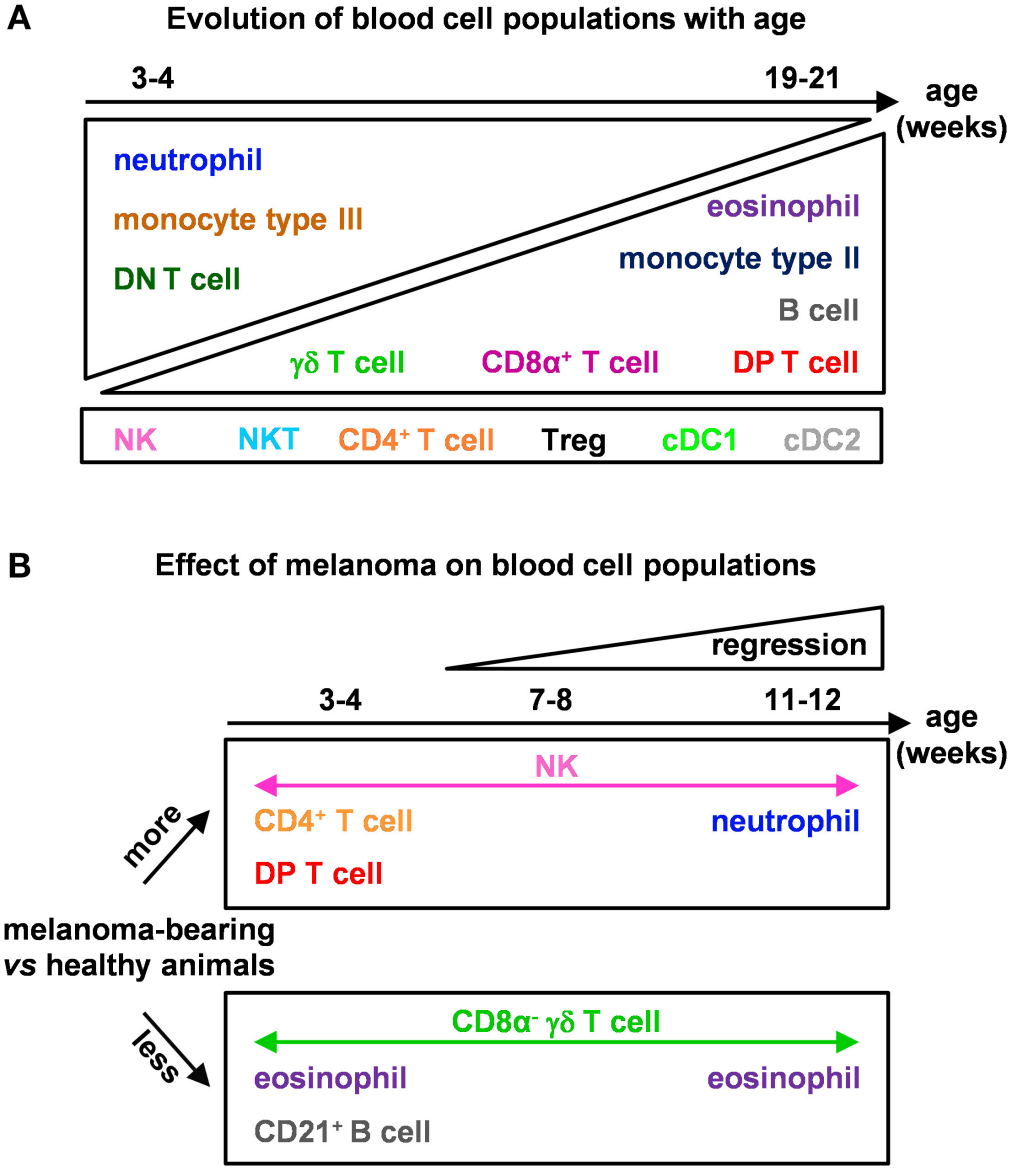
Models of the effects of age and melanoma occurrence on immune blood cell composition of MeLiM pigs. **(A)** Evolution of blood cell populations with age and **(B)** effect of melanoma on blood cell populations.

Here we showed major effects of age (Figure 7A) on B cells, γδ T cells, DP and CD8α^+^ T cells from total blood of 3–21 week-old MeLiM pigs, consistent with previous analyses on PBMCs in other breeds (29–31). In addition, we evidenced a decrease in neutrophils and an increase in eosinophils with age. The inter-individual variability in very young piglets may be related to a difference in the maturation of their immune system. For example, a high number of DN T cells was found in the youngest studied animals and should correspond to immature T cells. Also T and B lymphocytes are present in colostrum passing into neonate's blood and therefore could participate in this variability. For animal welfare, weaning is progressive in our experimental facility, piglets can start eating dry food while still suckling. Therefore, the effect of weaning could not be clearly investigated in this study.

We have refined the identification of granulocytes in swine. Indeed, these cells are mainly identified from the whole blood by their morphology (high granularity) or purified according to their density (32). The detailed identification of neutrophils, eosinophils and basophils was based on automatic blood cell count or by microscopy with a specific staining. We have defined a new strategy allowing the identification of eosinophils among granulocytes. They have a high granulometry (high SSC in log scale) and have no or low PG68A expression. This marker is currently considered as a pan granulocyte marker although its target is still unknown (9). We showed here that PG68A stains neutrophils and possibly basophils, but not eosinophils in swine blood. Being able for the first time to distinguish by flow cytometry eosinophils from neutrophils among granulocytes in pigs allowed us to reveal an inverse evolution between these two populations with age. Eosinophils are usually linked to parasite infestations. A careful follow up of animal health status was done and no parasitic presence was detected during the course of the study.

Monocyte subsets and their functionality are not clearly defined in pigs. Fairbairn et al. have reported variable expressions between breeds for CD163, CD14, and MHC II markers used to phenotype the four distinct subsets that could be more or less continuous (21). It seems difficult to compare from one study to another as the breed and age of pigs are different or not always reported. Nonetheless, two major subtypes are present in the blood of adult pigs: type I (CD163^−^ CD14^+^ MHC II^−^) and type IV (CD163^+^ CD14^low^ MHC II^+^) (33, 34). We report here variable proportions of monocytes with age. Globally, CD163^−^ monocytes increase, while CD163^+^ monocytes decrease with age. Furthermore, type III (CD163^+^ CD14^+^ MHC II^−^) monocytes are present in high proportion in blood of young piglets, whereas type I and IV are the major subtypes of monocytes in older pigs. Based on the literature, type I monocytes could be considered as the most immature stage and type IV as the most mature. Indeed, CD163^−^ monocytes could spontaneously switch their phenotype to CD163^+^ under *in vitro* conditions (24). In addition, CD163^+^ monocytes express higher amounts of co-stimulatory and adhesion molecules and are able to produce higher amounts of TNF-α than CD163^−^ ones after *in vitro* culture (23, 24). The approach of studying the morphological features of the different monocyte subsets by imaging flow cytometry could help understanding their activation status. We report here that type II monocytes display morphological features that correspond to a more advanced activation status (loss of cytoplasmic volume, high nucleus to cytoplasm ratio) (35) and that type III and IV monocytes are the biggest ones. It would be of great interest to use this approach to study monocytes in inflammatory infection conditions.

Recently, melanoma-bearing MeLiM pigs were reported to show white blood cell counts not significantly different from control MeLiM pigs, neutrophils instead were more abundant in sick ones (36). Here we confirm this effect on circulating neutrophils on growing animals. We further observed higher proportions of NK cells and lower proportions of eosinophils at all time points in melanoma-bearing animals (Figure 7B). At 3–4 weeks of age, these individuals also showed higher CD4^+^ and DP T cells, as well as lower percentages of γδ T cells and B cells. In line with our findings, the inoculation of melanoma cells in mice was reported to decrease B and CD8α ^+^ T lymphocytes, increase CD4^+^ T lymphocytes and NK cells percentages and activate markers of NK in blood (37). Patients with an advanced stage of melanoma have also higher circulating leukocytes, neutrophils and monocytes, and lower lymphocytes, supporting a shaping of the blood composition in the course of tumor progression (38).

Compared to human and mice, swine harbor a substantial proportion of γδ T cells in the peripheral blood and lymphoid organs like other species qualified as high γδ T species (17); their role is still unclear. In absolute count, CD8α^−^ γδ T cells are less frequent in melanoma-bearing pigs compared to healthy pigs. Nevertheless, in melanoma-bearing animals, we found a higher proportion and an equal absolute count of CD8α^+^ γδ T cells, which seems to stain more mature cells (39). B cells are also less abundant in melanoma-bearing animals than in healthy pigs, but higher proportion of CD21^−^ MHC II^+^ B cells among B cells is observed and absolute count of this specific B cell subset is maintained in animals with melanoma. In humans, CD21^−/low^ B cells increase with age in physiological conditions and correspond to memory B cells (40). In pigs, naïve and primed/activated B cells express CD21 while effector B cells and plasma cells are CD21^−^ (17, 41). Braun et al. have identified CD21^−^ B cells as B-1 cells (42). Here we found that CD21 expression on B cells correlated with the serum total IgG levels. Interestingly, Cizkova et al. also found an increase of melanoma-associated DP T lymphocytes in peripheral blood during melanoma regression at adult stages (43). In our study, we showed a higher proportion of this T cell subset already in young pigs. Together, all these B cell and T cell subsets may be involved in the production of tumor-specific antibodies and the depigmentation process associated with the regression (5). Furthermore, the higher proportion of NK cells in melanoma-bearing animals suggests an important implication of NK cells in the immune response against melanoma tumors in the MeLiM model. Interestingly, compared to healthy donors, an impaired blood NK cell function is observed in metastatic melanoma patients (44, 45). Accordingly, anti-tumor properties of NK cells and their subsequent role in the regression process remains to be addressed in MeLiM pigs. It could thus be envisaged to add NKp46 as a marker of NK cell activation (46).

The higher proportions of NK cells, CD4^+^ and DP T cells and CD21^−^ B cells among B cells in young melanoma-bearing piglets compared to healthy age matched pigs is in accordance with the mounting of an immune response against melanoma. The strategy designed here includes live/dead staining and an anti-CD45 antibody and could be adapted to identify cell subsets from more complex tissues in which dead cells can render the analysis more difficult and when identifying immune cells among others is necessary. Further studies are needed to characterize immune cells infiltrating melanoma tumors in this MeLiM biomedical model to better understand the role of the immune system in the progression and regression of melanomas. More knowledge on the phenotypes of immune cells in swine blood could also participate in qualifying and selecting immunocompetent pigs to improve welfare as well as to reduce antimicrobials in swine meat production.

## Data Availability Statement

The raw data supporting the conclusions of this article will be made available by the authors, without undue reservation, to any qualified researcher.

## Ethics Statement

The animal study was reviewed and approved by Committee on the Ethics of Animal Experiments of AgroParisTech and INRA Jouy-en-Josas (COMETHEA, authorization number 12/091).

## Author Contributions

FB, AP-B, NB, and SV-N contributed to the conception and design of the study. FB, GP, and EBoug performed the experiments. J-JL and FA were in charge of the animal experimentations. FB performed the statistical analysis and wrote the first draft of the manuscript. AP-B, GE, EBour, NB, and SV-N participated in interpreting the results. All authors contributed to manuscript revision, read and approved the submitted version.

### Conflict of Interest

The authors declare that the research was conducted in the absence of any commercial or financial relationships that could be construed as a potential conflict of interest.
