# Supplementary material for: The Composition of Circulating Leukocytes Varies With Age and Melanoma Onset in the MeLiM Pig Biomedical Model
Source: Front Immunol. 2020 Feb 28;11:291. doi: 10.3389/fimmu.2020.00291 (PMC7059855; doi:10.3389/fimmu.2020.00291)
Supplement: Supplementary file 1 [file Data_Sheet_1.docx]

Supplementary Material

**Supplementary Table 1: Absolute numbers and proportions of NK and T lymphocytes subsets in pig blood, and effect of age, sex and melanoma occurrence on those phenotypes.** Means, SD and 95% CI of mean obtained for the 36 pigs sampled from 3 to 21 weeks (n=161). Effects of age, sex and melanoma occurrence were calculated by likelihood ratio tests. All significant results are in bold.

| **cells** (phenotype) | **Mean** | **SD** | **95% CI of mean** | | **unit** | **(*p*) effects of** | | |
| --- | --- | --- | --- | --- | --- | --- | --- | --- |
|  |  |  | **lower** | **upper** |  | **age** | **sex** | **melanoma** |
| **NK cells** | 424 | 396 | 363 | 486 | 10^3^ cells / mm^3^ | 0.536 | 0.417 | **0.001** |
| (CD3^-^ CD8α^+^ CD16^+^) | 3.2 | 2.3 | 2.8 | 3.5 | % of PBLs | 0.291 | 0.198 | **< 0.001** |
| **T cells** | 3299 | 1404 | 3080 | 3517 | 10^3^ cells / mm^3^ | **0.002** | 0.811 | **0.005** |
| (CD3^+^) | 25.6 | 9 | 24.2 | 27 | % of PBLs | **0.001** | 0.465 | **0.008** |
| **NKT cells** | 47.2 | 39.5 | 41.1 | 53.4 | 10^3^ cells / mm^3^ | **< 0.001** | 0.185 | 0.285 |
| (CD3^+^ TCR1 δ chain^-^ CD16^+^) | 0.4 | 0.3 | 0.3 | 0.4 | % of PBLs | **< 0.001** | 0.085 | 0.974 |
|  | 1.4 | 1.1 | 1.3 | 1.6 | % of T cells | **< 0.001** | 0.148 | 0.263 |
| **γδ T cells** | 1281 | 824 | 1153 | 1410 | 10^3^ cells / mm^3^ | **0.009** | 0.795 | **< 0.001** |
| (CD3^+^ TCR1 δ chain^+^) | 9.9 | 6 | 9 | 10.9 | % of PBLs | **< 0.001** | 0.926 | **< 0.001** |
|  | 37.2 | 13.6 | 35.1 | 39.3 | % of T cells | **0.002** | 0.511 | **< 0.001** |
| **CD8α^+^ γδ T cells** | 14.1 | 7.5 | 12.9 | 15.3 | % of γδ T cells | **< 0.001** | 0.082 | **< 0.001** |
| (CD3^+^ TCR1 δ chain^+^ CD8α^+^) |  |  |  |  |  |  |  |  |
| **CD4^+^ CD8α^-^ T helper** | 833 | 436 | 765 | 900 | 10^3^ cells / mm^3^ | **< 0.001** | 0.057 | 0.174 |
| (CD3^+^ TCR1 δ chain^-^ CD16^-^ CD4^+^ CD8α^-^) | 6.6 | 3.3 | 6.1 | 7.1 | % of PBLs | 0.083 | **0.017** | **0.047** |
|  | 26.8 | 11.5 | 25 | 28.6 | % of T cells | **< 0.001** | **0.014** | **< 0.001** |
| **CD4^+^ CD8α^+^ T cells** | 255 | 140 | 233 | 277 | 10^3^ cells / mm^3^ | **< 0.001** | 0.914 | **0.001** |
| (CD3^+^ TCR1 δ chain^-^ CD16^-^ CD4^+^ CD8α^+^) | 2 | 1.1 | 1.8 | 2.2 | % of PBLs | **< 0.001** | 0.579 | **< 0.001** |
|  | 8.3 | 4.2 | 7.6 | 8.9 | % of T cells | **< 0.001** | 0.860 | **< 0.001** |
| **CD4^-^ CD8α^+^ cytotoxic T cells** | 685 | 395 | 623 | 746 | 10^3^ cells / mm^3^ | **0.003** | 0.064 | 0.115 |
| (CD3^+^ TCR1 δ chain^-^ CD16^-^ CD4^-^ CD8α^+^) | 5.2 | 2.4 | 4.9 | 5.6 | % of PBLs | **0.002** | **0.040** | 0.216 |
|  | 20.6 | 6.8 | 19.6 | 21.7 | % of T cells | 0.151 | **< 0.001** | 0.220 |
| **CD4^-^ CD8α^-^ T cells** | 199 | 250 | 160 | 238 | 10^3^ cells / mm^3^ | **< 0.001** | 0.357 | **0.013** |
| (CD3^+^ TCR1 δ chain^-^ CD16^-^ CD4^-^ CD8α^-^) | 1.5 | 1.7 | 1.2 | 1.8 | % of PBLs | **< 0.001** | 0.328 | 0.058 |
|  | 5.7 | 6 | 4.8 | 6.6 | % of T cells | **< 0.001** | 0.390 | 0.283 |
| **Tregs** | 124 | 56.8 | 115 | 133 | 10^3^ cells / mm^3^ | **0.006** | 0.992 | 0.915 |
| (CD3^+^ CD25^+^ Foxp3^+^) | 1 | 0.3 | 0.9 | 1 | % of PBLs | 0.632 | 0.669 | 0.540 |
|  | 3.9 | 1 | 3.7 | 4 | % of T cells | **< 0.001** | 0.706 | **< 0.001** |
| **CD4^+^ CD8α^-^ Tregs** | 63.7 | 9.9 | 62.2 | 65.3 | % of Tregs | 0.108 | 0.925 | **< 0.001** |
| (CD3^+^ CD25^+^ Foxp3^+^ CD4^+^ CD8α^-^) |  |  |  |  |  |  |  |  |
| **CD4^+^ CD8α^+^ Tregs** | 16.8 | 7.8 | 15.6 | 18 | % of Tregs | **< 0.001** | 0.252 | 0.155 |
| (CD3^+^ CD25^+^ Foxp3^+^ CD4^+^ CD8α^+^) |  |  |  |  |  |  |  |  |
| **CD4^-^ CD8α^+^ Tregs** | 12.5 | 5.24 | 11.7 | 13.3 | % of Tregs | **0.041** | **0.033** | **< 0.001** |
| (CD3^+^ CD25^+^ Foxp3^+^ CD4^-^ CD8α^+^) |  |  |  |  |  |  |  |  |
| **CD4^-^ CD8α^-^ Tregs** | 6.9 | 3.4 | 6.4 | 7.5 | % of Tregs | 0.527 | 0.555 | **0.008** |
| (CD3^+^ CD25^+^ Foxp3^+^ CD4^-^ CD8α^-^) |  |  |  |  |  |  |  |  |

**Supplementary Table 2: Absolute numbers and proportions of the B cells in pig blood, and effect of age, sex and melanoma occurrence on those phenotypes.** Means, SD and 95% CI of mean obtained for the 36 pigs sampled from 3 to 21 weeks (n=161). Effects of age, sex and melanoma occurrence were calculated by likelihood ratio tests. All significant results are in bold.

| **cells** (phenotype) | **Mean** | **SD** | **95% CI of mean** | | **unit** | **(*p*) effects of** | | |
| --- | --- | --- | --- | --- | --- | --- | --- | --- |
|  |  |  | **lower** | **upper** |  | **age** | **sex** | **melanoma** |
| **B cells** | 928 | 625 | 831 | 1025 | 10^3^ cells / mm^3^ | **< 0.001** | 0.392 | **0.013** |
| (CD79a^+^) | 6.9 | 3.6 | 6.3 | 7.4 | % of PBLs | **< 0.001** | 0.570 | **0.008** |
| **MHC II^+^ CD21^+^ B cells** | 78.8 | 7.1 | 77.7 | 79.9 | % of B cells | **< 0.001** | 0.398 | **< 0.001** |
| (CD179a^+^ MHC II^+^ CD21^+^) |  |  |  |  |  |  |  |  |
| **MHC II^+^ CD21^-^ B cells** | 17.3 | 6.3 | 16.3 | 18.8 | % of B cells | **< 0.001** | 0.894 | **< 0.001** |
| (CD179a^+^ MHC II^+^ CD21^-^) |  |  |  |  |  |  |  |  |
| **MHC II^-^ CD21^-^ B cells** | 3.6 | 2.8 | 3.1 | 4 | % of B cells | **0.001** | 0.320 | 0.067 |
| (CD179a^+^ MHC II^-^ CD21^-^) |  |  |  |  |  |  |  |  |

**Supplementary Table 3: Absolute numbers and proportions of the different myeloid cell subsets in pig blood, and effect of age, sex and melanoma occurrence on those phenotypes.** Means, SD and 95% CI of mean obtained for the 36 pigs sampled from 3 to 21 weeks (n=161, except for monocytes where n=153). Effects of age, sex and melanoma occurrence were calculated by likelihood ratio tests. All significant results are in bold.

| **cells** (phenotype) | **Mean** | **SD** | **95% CI of mean** | | **unit** | **(*p*) effects of** | | |
| --- | --- | --- | --- | --- | --- | --- | --- | --- |
|  |  |  | **lower** | **upper** |  | **age** | **sex** | **melanoma** |
| **cDC1** | 3.8 | 3.8 | 3.2 | 4.3 | 10^3^ cells / mm^3^ | **< 0.001** | 0.106 | 0.814 |
| (FSC^med^ SSC^med^ MHC II^high^ CD14^-^ CD163^-/low^ CADM1^high^ CD172a^med^) | 0.03 | 0.02 | 0.02 | 0.03 | % of PBLs | **< 0.001** | 0.128 | 0.607 |
| **cDC2** | 5.5 | 6.7 | 4.4 | 6.5 | 10^3^ cells / mm^3^ | **0.049** | 0.095 | 0.054 |
| (FSC^med^ SSC^med^ MHC II^high^ CD14^-^ CD163^-/low^ CADM1^med^ CD172a^high^) | 0.04 | 0.05 | 0.03 | 0.05 | % of PBLs | **0.005** | **0.030** | **0.021** |
| **CD163^-^ monocytes** | 1001 | 500 | 923 | 1079 | 10^3^ cells / mm^3^ | **0.008** | 0.911 | 0.517 |
| (FSC^med^ SSC^med^ CD172a^+^ CD163^-^) | 7.3 | 2.0 | 7.0 | 7.6 | % of PBLs | **0.010** | 0.571 | 1.000 |
|  | 60.2 | 12.2 | 58.3 | 62.1 | % of monocytes | **< 0.001** | 0.114 | 0.832 |
| **CD163^+^ monocytes** | 719 | 641 | 619 | 819 | 10^3^ cells / mm^3^ | **< 0.001** | 0.197 | 0.690 |
| (FSC^med^ SSC^med^ CD172a^+^ CD163^+^) | 5.0 | 2.4 | 4.6 | 5.4 | % of PBLs | **< 0.001** | **0.017** | 0.895 |
|  | 39.8 | 12.2 | 37.9 | 41.8 | % of monocytes | **< 0.001** | 0.124 | 0.780 |
| **Type I monocytes** | 499 | 285 | 454 | 545 | 10^3^ cells / mm^3^ | **0.013** | 0.624 | 0.555 |
| (FSC^med^ SSC^med^ CD172a^+^ | 3.7 | 1.3 | 3.5 | 3.9 | % of PBLs | **0.012** | 0.755 | 0.856 |
| CD163^-^ CD14^+^ MHC II^-^) | 30.7 | 9 | 29.3 | 32.2 | % of monocytes | **< 0.001** | 0.062 | 0.831 |
| **Type II monocytes** | 349 | 256 | 308 | 390 | 10^3^ cells / mm^3^ | **0.004** | 0.675 | 0.463 |
| (FSC^med^ SSC^med^ CD172a^+^ | 2.6 | 1.4 | 2.3 | 2.8 | % of PBLs | **0.006** | 0.366 | 0.938 |
| CD163^-^ CD14^low^ MHC II^+^) | 21.1 | 10 | 19.5 | 22.7 | % of monocytes | **0.001** | 0.960 | 0.943 |
| **Type III monocytes** | 151 | 251 | 111 | 191 | 10^3^ cells / mm^3^ | **< 0.001** | 0.952 | 0.092 |
| (FSC^med^ SSC^med^ CD172a^+^ | 1 | 1.2 | 0.8 | 1.2 | % of PBLs | **< 0.001** | 0.372 | 0.650 |
| CD163^+^ CD14^+^ MHC II^-^) | 8.2 | 8.8 | 6.8 | 9.6 | % of monocytes | **< 0.001** | 0.719 | 0.608 |
| **Type IV monocytes** | 457 | 264 | 414 | 499 | 10^3^ cells / mm^3^ | 0.263 | 0.285 | 0.880 |
| (FSC^med^ SSC^med^ CD172a^+^ | 3.4 | 1.3 | 3.2 | 3.6 | % of PBLs | 0.738 | **0.021** | 0.208 |
| CD163^+^ CD14^low^ MHC II^+^) | 28.2 | 8.11 | 26.9 | 29.5 | % of monocytes | 0.061 | 0.180 | 0.123 |
| **Neutrophils** | 7150 | 4426 | 6461 | 7839 | 10^3^ cells / mm^3^ | **< 0.001** | 0.374 | 0.698 |
| (FSC^med^ SSC^high^ PG68A^+^) | 50 | 11.1 | 48.3 | 51.7 | % of PBLs | **< 0.001** | 0.093 | **0.010** |
| **Eosinophils** | 245 | 214 | 211 | 278 | 10^3^ cells / mm^3^ | **< 0.001** | 0.367 | **0.003** |
| (FSC^med^ SSC^high^ PG68A^-/low^) | 1.8 | 1.2 | 1.6 | 2 | % of PBLs | **< 0.001** | 0.660 | **0.001** |


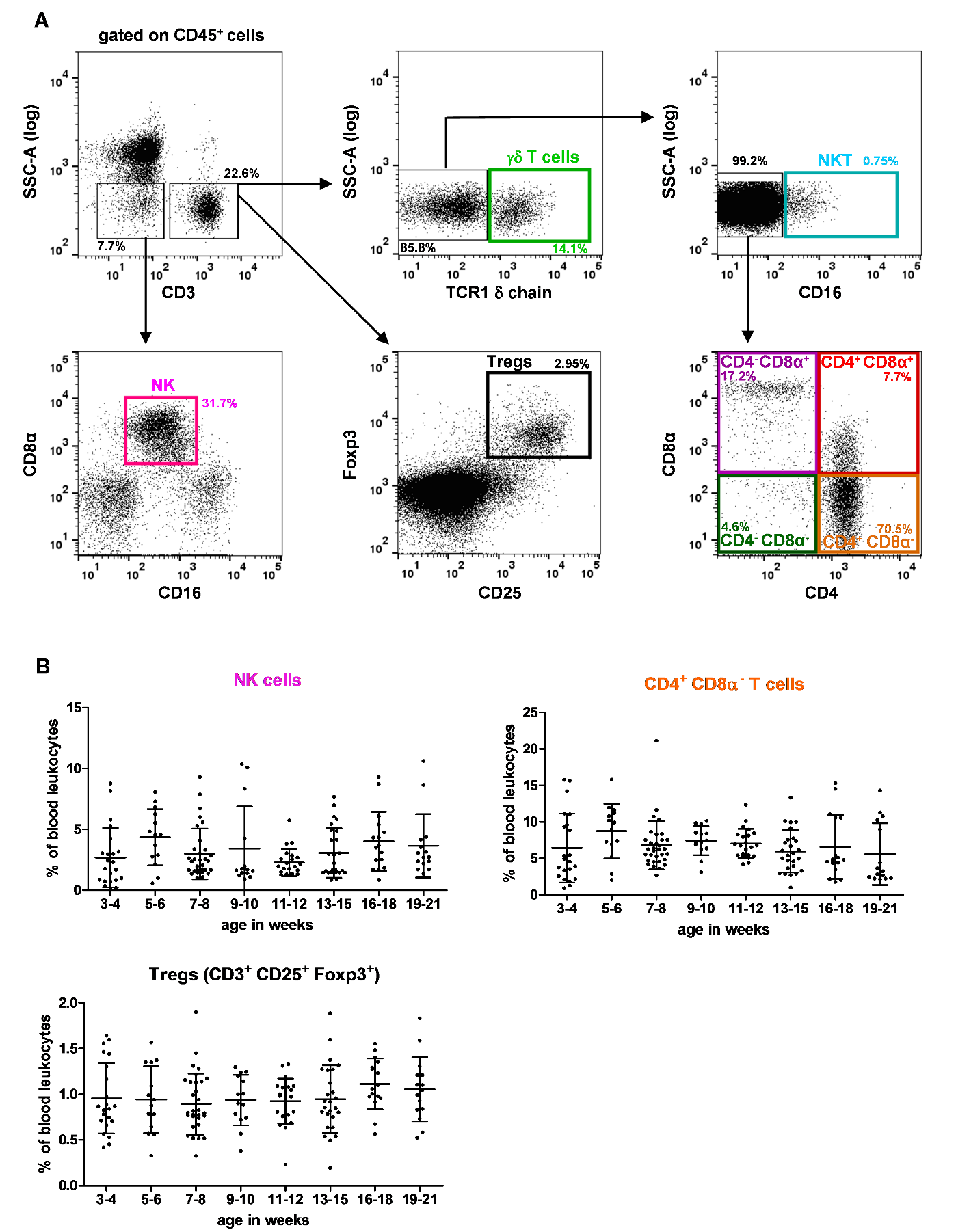


**Supplementary Figure 1: NK, γδ T cells, CD4 T helper, cytotoxic CD8 T lymphocytes and Tregs in swine peripheral blood. (A)** Gating strategy to identify NK, NKT, γδ T, CD4 T helper, cytotoxic CD8 T lymphocytes and Tregs in PBLs. Cells were first gated on the CD45^+^ population described in Figure 1A. CD3^+^ and CD3^-^ cells were gated (upper left panel). NK cells were identified as CD8α^+^ CD16^+^ cells within the CD3^-^ gate (lower left panel). Among CD3^+^ T cells, γδ T cells were characterized by their expression of TCR1 δ chain (upper middle panel). CD3^+^ T cells, not expressing TCR1 δ chain, were then analyzed for their expression of CD16 (upper right panel). CD3^+^ TCR1 δ chain^-^ CD16^+^ cells corresponded to NKT cells. Among CD3^+^ TCR1 δ chain^-^ CD16^-^ cells, CD4 and CD8α expressions were finally analyzed (lower right panel): CD4^+^ CD8α^+/-^ corresponded to CD4 T helper cells and CD4^-^ CD8α^+^ to cytotoxic T lymphocytes. Tregs were defined as CD25^+^ Foxp3^+^ among CD3^+^ T cells (lower middle panel). Percentages of the parent populations are shown on each dot plot. In all panels, 10000 events are represented except for NKT cells’ gating where 100000 events are represented. **(B)** Kinetics of the percentages of NK, CD4^+^ CD8α^-^ T lymphocytes and Tregs in PBLs. Lines represent the means with SEM. Significant differences are represented with bars (* *p* < 0.05, ** *p* < 0.01 and *** *p* < 0.001).

**
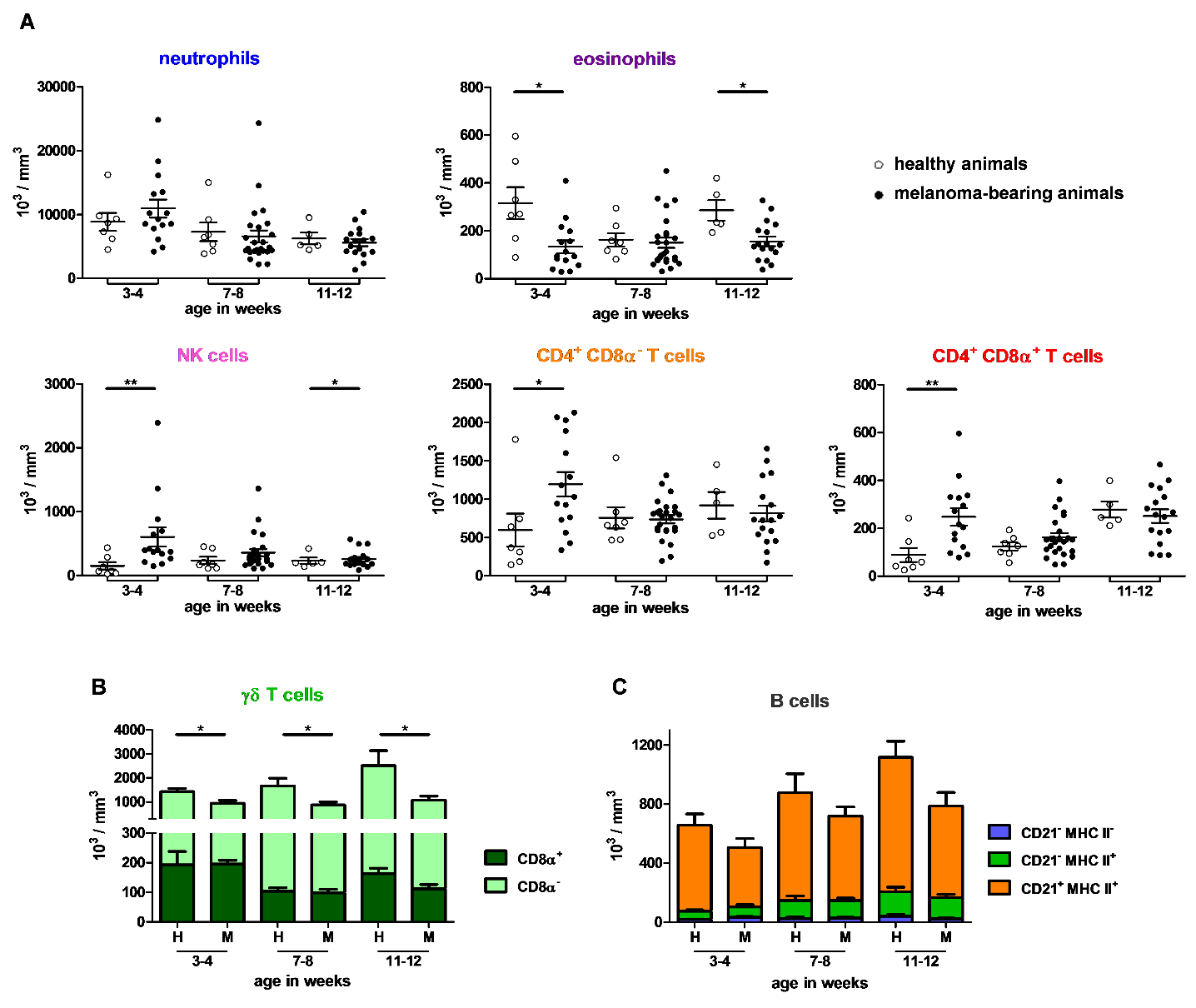
**

**Supplementary Figure 2: Effect of melanoma occurrence on immune blood cell counts of MeLiM pigs.** (**A**) Absolute count number of neutrophils, eosinophils, NK cells, CD4^+^ CD8α^-^ T cells and CD4^+^ CD8α^+^ T cells in blood from healthy and melanoma-bearing pigs aged of 3-4, 7-8 and 11-12 weeks. Absolute count number (**B**) of γδ T cells depending on their expression of CD8α and (**C**) of B cells depending on their expressions of CD21 and MHC II in blood from healthy (H) and melanoma-bearing (M) pigs aged of 3-4, 7-8 and 11-12 weeks. Lines represent the means with SEM. Healthy and melanoma-bearing pigs at each age were compared using a Mann-Whitney test and significant differences are represented with bars (* *p* < 0.05).
